# Artificial Cochlear Sensory Epithelium with Functions of Outer Hair Cells Mimicked Using Feedback Electrical Stimuli

**DOI:** 10.3390/mi9060273

**Published:** 2018-05-30

**Authors:** Tetsuro Tsuji, Asuka Nakayama, Hiroki Yamazaki, Satoyuki Kawano

**Affiliations:** Graduate School of Engineering Science, Osaka University, Toyonaka, Osaka 560-8531, Japan; tsuji@me.es.osaka-u.ac.jp (T.T.); nakayama@bnf.me.es.osaka-u.ac.jp (A.N.); yamazaki@bnf.me.es.osaka-u.ac.jp (H.Y.)

**Keywords:** artificial cochlea, MEMS, piezoelectric material, outer hair cell

## Abstract

We report a novel vibration control technique of an artificial auditory cochlear epithelium that mimics the function of outer hair cells in the organ of Corti. The proposed piezoelectric and trapezoidal membrane not only has the acoustic/electric conversion and frequency selectivity of the previous device developed mainly by one of the authors and colleagues, but also has a function to control local vibration according to sound stimuli. Vibration control is achieved by applying local electrical stimuli to patterned electrodes on an epithelium made using micro-electro-mechanical system technology. By choosing appropriate phase differences between sound and electrical stimuli, it is shown that it is possible to both amplify and dampen membrane vibration, realizing better control of the response of the artificial cochlea. To be more specific, amplification and damping are achieved when the phase difference between the membrane vibration by sound stimuli and electrical stimuli is zero and π, respectively. We also demonstrate that the developed control system responds automatically to a change in sound frequency. The proposed technique can be applied to mimic the nonlinear response of the outer hair cells in a cochlea, and to realize a high-quality human auditory system.

## 1. Introduction

Hearing is important to infants in terms of acquiring language and sentiment education. However, one in a thousand new-born infants suffers congenital deafness. Meanwhile, the ability of adults to hear diminishes gradually and never recovers spontaneously. There is thus an increasing demand for artificial cochleae, which are devices that support patients with sensorineural hearing loss. The present study deals with the development of a novel technique that allows the design of a higher-quality artificial cochlea to be combined with previous devices developed by one of the authors [1,2,3].

The vibration of sound is collected and amplified by outer and middle ears, and transmitted to the inner ear. The frequency of sound is then distinguished by cochlear epithelium in the inner ear [4], and the vibration of sound is converted into electrical signals by inner hair cells to stimulate nerves, which are sensory cells on the epithelium. Because damaged hair cells do not regenerate spontaneously [5], cochlear implants [6,7] are used to effectively overcome sensorineural hearing loss due to cochlear damage . However, commercially available cochlear-implant devices require patients to wear external equipment such as microphones, sound processors, and batteries. To remove such burdens on patients and to increase patients’ quality of life, a self-contained artificial cochlea has been proposed by one of the authors and their colleagues [1,2,3]. Recent advances in the fabrication technologies of micro-electro-mechanical systems have allowed the development of an artificial cochlear epithelium without an external power supply [1,2,8,9,10,11,12,13,14,15,16,17,18,19]. Electrical signals in these devices are generated by the deformation of a trapezoidal piezoelectric membrane induced by sound stimuli. Such a mechanical generation of electrical signals has been proposed as an energy harvester [20] or flow sensor [21] in biological systems, and its performance as a micro-electro-mechanical-system-fabricated acoustic transducer has been quantitatively investigated [22,23]. Moreover, a method of fixing the device in the cochlea has also been proposed by one of the authors [24] to show the feasibility of the artificial cochlear epithelium made of piezoelectric materials.

In addition to having frequency selectivity and converting vibration to electrical signals, the cochlea amplifies signals through active feedback [25,26,27]. In fact, the cochlear basilar membrane has a nonlinear response to sound stimuli, and this response is attributed to the functions of the outer hair cells in the organ of Corti [28,29,30,31,32,33]; that is, the outer hair cells have the function of controlling the sound level to be recognized. To be more specific, a weak sound input is amplified and a strong input is damped. Thanks to these nonlinear responses, human hearing has a wide dynamic range from 0 to 120 dB in amplitude, although the range of displacement of the cochlear epithelium is from 0.1 to 10 nm [30]. Since the nonlinear response is partly realized by the mechanical feature of the basilar membrane [30], it is important to mimic this feature in engineering the artificial cochlea. In fact, an attempt to mimic the nonlinear response has been made using a cantilever model with dimensions of 470 × 38 mm [34]. It is shown in Reference [34] that a cubic damping term with respect to the cantilever velocity in the control signal realizes the nonlinear response of the cantilever velocity against a disturbance. Moreover, the nonlinear response had similar characteristics with human hearing.

The present paper reports the development of an artificial cochlear epithelium which controls the local vibration according to sound stimuli, on the basis of our preceding study [2]. To be more specific, the preceding study [2] mimicked the function of the basilar membrane and inner hair cells, while the present study also mimics the function of outer hair cells. The development of such a control technique is expected to produce a device that realizes the nonlinear response. Moreover, the control technique can be used to better understand the cochlear mechanism by providing an experimental method that replicates the cochlear behavior.

The artificial cochlear epithelium in the present study is a trapezoidal piezoelectric membrane with patterned Al electrodes [2], where each electrode has two small Al plates for the electrical output and feedback input. The resonance frequency of the device varies locally according to the longitudinal position, achieving the function of frequency selectivity. The patterned electrodes have two functions. One is to recognize sound stimuli through the piezoelectric effect induced by the local deformation of the membrane, while the other is to control local vibration through the inverse piezoelectric effect by applying external electrical stimuli. It is found that the local membrane vibration can be amplified or damped by choosing appropriate phase differences between the electrical and sound stimuli. Using these amplification and damping controls, the nonlinear response that partly realizes the wide dynamic range of human hearing is obtained. Such techniques for recognition and control can be applied to the development of the artificial cochlear epithelium, which mimics functions of the basilar membrane, inner hair cells, and outer hair cells. These functions are needed to achieve the high performance of the human cochlea, having a wide range frequency selectivity from 20 to 20,000 Hz and a wide dynamic range from 0 to 120 dB.

## 2. Experimental Method

### 2.1. Fabrication of the Artificial Cochlear Epithelium

**Figure 1:**
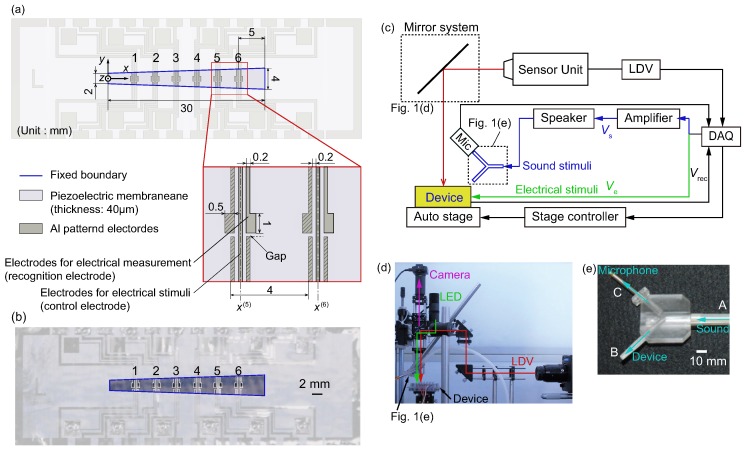
(**a**) Schematic and (**b**) photograph of the artificial cochlear epithelium fabricated in this paper. The patterned electrodes were fabricated on a polyvinylidene difluoride (PVDF) piezoelectric membrane with thickness of 40 µm; (**c**) Overview of the experimental setup. The multichannel analog output module in the data acquisition (DAQ) system was used to apply the sound and electrical stimuli with a desired phase difference. The DAQ system was also used to analyze the electrical output from the device and the results of laser Doppler vibrometer (LDV) measurement. These analyzed data were used in feedback control; (**d**) Details of the mirror system in panel (c) to observe the exact position of the laser for LDV measurement; (**e**) Photograph of an acyclic part used to fix the microphone.

We first give an overview of the developed device. We use a piezoelectric membrane having a fixed boundary conditions with a trapezoidal shape as shown in Figure 1a. To control the vibration of the membrane, we apply electrical stimuli to patterned electrodes fabricated on the piezoelectric membrane. The patterned electrodes can also be used to recognize the vibration amplitude. Note that in previous studies [1,2], the electrodes were used to generate the electrical signals to stimulate nerves, mimicking the function of the inner hair cells. In the present study, however, the electrodes are used for feedback control to also mimic the function of the outer hair cells. The vibrations induced by sound and electrical stimuli are expected to be superposed. The piezoelectric material used in this study is a polyvinylidene difluoride (PVDF), because PVDF is biologically compatible [8]. We describe the fabrication method and details of the device in the following.

The device has patterned Al electrodes, as shown in Figure 1a,b. Electrodes have two functions, and are referred to as recognition and control electrodes accordingly. Six pairs of recognition and control electrodes are fabricated on the PVDF membrane. The backside of the PVDF membrane is completely covered by Al deposition and is electrically grounded. The number of electrode pairs was chosen so that local amplitude control can be demonstrated while minimizing the complexity of the device.

The Cartesian coordinate system is defined as in Figure 1a, and the *x*-coordinates of the center of the *i*-th control electrode, $x^{\left(i\right)}$ (i=1,2,⋯,6), are set as $x^{\left(1\right)}=5$ mm, $x^{\left(2\right)}=9$ mm, $x^{\left(3\right)}=13$ mm, $x^{\left(4\right)}=17$ mm, $x^{\left(5\right)}=21$ mm, and $x^{\left(6\right)}=25$ mm. The dimensions of the recognition electrodes (0.5 × 1.0 mm centered at y=0 mm) are the same as those in our previous study [2], while those of control electrodes are smaller (0.2 mm) to achieve a better localization of electrical stimuli. The distance between recognition and control electrodes is 0.2 mm. This value of distance was chosen to be small in order to apply electrical stimuli near the recognition electrodes, but the distance should be large enough to achieve a stable electrode-patterning process on the PVDF membrane. The electrode pattern is designed so that geometrical symmetry is preserved but the main feature of the recognition electrode is the same as that in our previous study [2]. To be more precise, the pattern is symmetric with respect to the *x*-axis, except for the small gap indicated in the magnified figure of Figure 1a. The electrodes with diagonal lines are prepared to have geometrical symmetry, and were not used in this study.

The device was fabricated by attaching the PVDF membrane to a stainless-steel plate with a trapezoidal slit using double-sided tape (No. 500, Nitto Denko, Osaka, Japan). The fabrication process is summarized in the Appendix. The method of assembly was carefully chosen so that the device will not degrade with time. The slit has length $L_{x}=30$ mm in the *x*-direction, and short and long sides in the *y*-direction are $L_{y,\mathrm{short}}$ = 2 mm and $L_{y,\mathrm{long}}$ = 4 mm in length, as shown in Figure 1a. The length in the *y*-direction of the trapezoidal membrane is therefore expressed as $l\left(x\right)=L_{y,\mathrm{short}}+\left(L_{y,\mathrm{long}}-L_{y,\mathrm{short}}\right)\left(x/L_{x}\right)$. These values are the same as those in Reference [2].

### 2.2. Experimental Setup

An overview of the experimental setup is shown in Figure 1c. The data acquisition (DAQ) system consists of a controller (NI PXIe-1082, NI PIXIe-8840, National Instruments, Austin, TX, USA), multichannel analog output module (NI PIXIe-6738, National Instruments, Austin, TX, USA) for sound and electrical stimuli, and a vibration module (NI PIXIe-6738, National Instruments, Austin, TX, USA) for data analysis. The device was fixed on an *x*–*y* auto-stage, which was also controlled by the DAQ system. A laser Doppler vibrometer (LDV; AT3600, AT0023, Graphtec, Tokyo, Japan) was used to measure the vibration of the device. The laser was irradiated through a mirror system (Figure 1d), where a camera (DCC1645C, Thorlabs, Newton, NJ, USA) and light-emitting diode as a light source (LEDD18/M565L3, Thorlabs, Newton, NJ, USA) were installed to precisely observe the position of the laser (i.e., the measuring point). The electrical signal $V_{s}$ from the DAQ system was magnified by an amplifier (HSA4-14, NF, Yokohama, Japan ) and applied to a speaker (FX102, Fostex, Tokyo, Japan). Here, $V_{s}$ was set to $V_{s}={\bar{V}}_{s}\sin\left(2\pi f_{s}t\right)$, where ${\bar{V}}_{s}$, $f_{s}$, and *t* are respectively the voltage amplitude, frequency of sound stimuli, and time variable. A microphone (377C01, 426B03, 480C02, Piezotronics, Depew, NY, USA) was used to evaluate the magnitude of the sound stimuli, $P_{s}$ (dB SPL). To measure $P_{s}$ near the membrane, we fabricated the acyclic part as shown in Figure 1e. Sound came from A in Figure 1e through a tube connected to a speaker, and passed to B (the device) and C (the microphone). The distance between point B and the membrane was set to less than 4 mm. The electrical signal $V_{e}^{\left(i\right)}$ from the DAQ system was applied to the *i*-th control electrode, where

(1) $$V_{e}^{\left(i\right)}={\bar{V}}_{e}^{\left(i\right)}\sin\left(2\pi f_{e}^{\left(i\right)}t+\phi^{\left(i\right)}\right),\;\left(i=1,2,\cdots ,6\right).$$

The parameter $\phi^{\left(i\right)}$ is the phase difference between the signals for sound and electrical stimuli. Details are given in Section 3.2. The electrical outputs $V_{\mathrm{rec}}^{\left(i\right)}$ from the *i*-th recognition electrodes and their amplitudes ${\bar{V}}_{\mathrm{rec}}^{\left(i\right)}$ were stored in the DAQ system.

## 3. Results and Discussion

### 3.1. Frequency Selectivity

**Figure 2:**
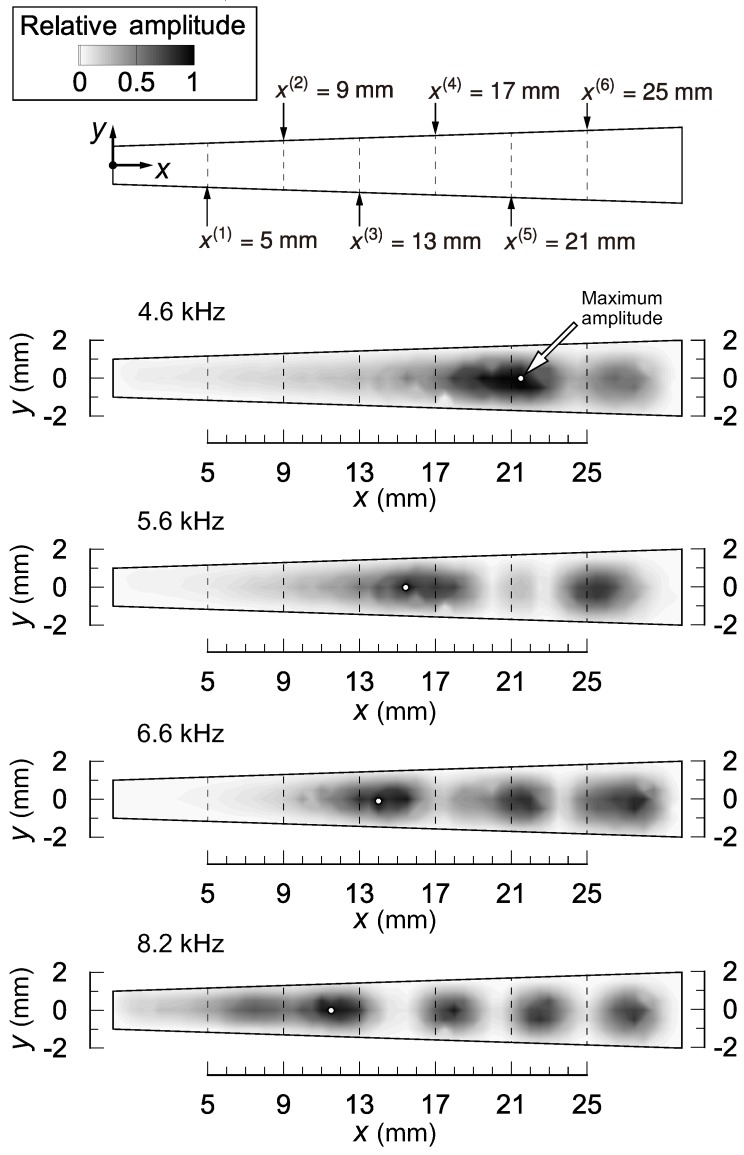
Amplitude distribution of membrane vibration in the *x*–*y* plane induced by sound stimuli with $f_{s}$ = 4.6, 5.6, 6.6, and 8.2 kHz. Contours show the relative amplitude normalized by the maximum value for each $f_{s}$. The resonant positions, at which the maximum value was obtained, are indicated by white dots. For each $f_{s}$, the resonant positions were different, and we thus conclude that the present device had frequency selectivity. Owing to the vibration mode in the *x*-direction, a couple of peaks were observed for each $f_{s}$. This tendency is the same as that observed for a previous device [2] developed by one of the authors.

First of all, we will briefly present the function of frequency selectivity of the present device. More detailed investigations of the function of frequency selectivity (e.g., a comparison with the Wentzel–Kramers–Brillouin (WKB) asymptotic solution and the effect of the surrounding fluid) were conducted in our previous study [2]. Figure 2 shows the amplitude distribution of membrane vibration in the *x*–*y* plane. Here, the vibration was induced by sound stimuli with $f_{s}$ = 4.6, 5.6, 6.6, and 8.2 kHz. These frequencies are close to the resonant frequencies of second, third, fourth, and fifth electrodes, respectively, which will be presented in Section 3.2.1. Contours show the relative amplitude normalized by the maximum value for each $f_{s}$. The resonant positions, at which the maximum value is obtained, are indicated by white dots. For each $f_{s}$, the resonant positions were different, and we thus conclude that the present device has frequency selectivity. Owing to the vibration mode in the *x*-direction and/or higher frequency modes, a couple of peaks were observed for each $f_{s}$. This tendency became prominent as $f_{s}$ increased and was the same as that of the previous device [2], but is not observed in the mammalian cochlear epithelium [33]. The duplication of the full details of the vibration modes observed in the mammalian cochlear epithelium will require the geometry and boundary condition to be mimicked. This difficulty is inherent to the membrane vibration, and its solution is our ultimate goal of future research. The development of a device that has simpler vibration modes and a clear one-to-one relation between the resonant position and frequency will help provide a solution.

### 3.2. Local Vibration Control Using Electrical Stimuli

This section describes the results of local vibration control using electrical stimuli. Section 3.2.1 determines parameters needed for vibration control, while Section 3.2.2 and Section 3.2.3 present examples of local vibration control.

#### 3.2.1. Search for Resonant Frequencies and Control Parameters

We first show that electrical stimuli induce vibration of the membrane and can be used to determine resonant frequencies $f_{\mathrm{res}}^{\left(i\right)}$ at position $x^{\left(i\right)}$ of the control electrodes by changing $f_{e}^{\left(i\right)}$ in Equation (1) continuously. Note that we measured the vibration of the membrane using an LDV and obtained the amplitude $a_{f}$ for the frequency component *f* through Fourier analysis.

**Figure 3:**
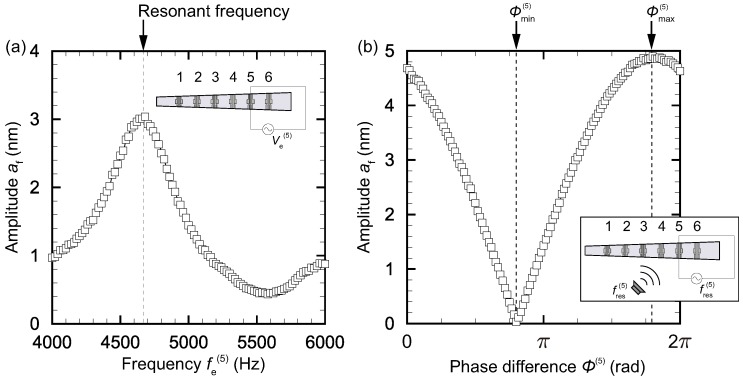
(**a**) Vibration amplitude at the fifth electrode (x=21 mm) for electrical stimuli applied to the fifth electrode with frequency $f_{e}^{\left(5\right)}$. The resonant frequency can be determined by sweeping the frequency $f_{e}^{\left(5\right)}$ in Equation (1); (**b**) Phase dependence on the vibration amplitude at the fifth electrode under sound stimuli with $f_{s}=f_{\mathrm{res}}^{\left(5\right)}$ and electrical stimuli with $f_{e}^{\left(5\right)}=f_{\mathrm{res}}^{\left(5\right)}$. The amplitude is at a maximum and $a_{f}\approx 5$ nm for a certain phase $\phi^{\left(5\right)}=\phi_{\max}^{\left(5\right)}\approx 1.8\pi$, while the amplitude is at a minimum and $a_{f}\approx 0$ nm for an another phase $\phi^{\left(5\right)}=\phi_{\min}^{\left(5\right)}\approx 0.8\pi$. The appropriate choice of $\phi^{\left(i\right)}$ leads to amplification or damping control of the membrane vibration.

Figure 3a shows the amplitude of membrane vibration on the fifth electrode (x=21 mm) induced by electrical stimuli applied to that electrode with different frequencies 4 kHz $\leq f_{e}^{\left(5\right)}\leq$ 6 kHz and ${\bar{V}}_{e}^{\left(5\right)}=5$ V. The amplitude reached a maximum at $f_{e}^{\left(i\right)}=4.66$ kHz, and we thus conclude that the resonant frequency at the position of the fifth electrode was $f_{\mathrm{res}}^{\left(5\right)}=4.66$ kHz. Before going into further detail, we will compare the experimental results with a theoretical prediction. Assume that the trapezoidal membrane can be considered as sequential beams of length l(x); that is, we neglect the vibration mode in the *x*-direction. Then, according to the linear theory of transverse vibration of a beam with fixed ends [35], we obtain the first-order resonant frequency as $f_{\mathrm{res}}^{\left(\mathrm{theory}\right)}=\left(22.37/2\pi \right){\left(EJ/\rho A\right)}^{1/2}/l^{2}\left(x\right)$, where *E* is the modulus of elasticity, ρ the mass density, A=hl(x) the cross-sectional area with *h* the thickness of the membrane, and $J=h^{3}l\left(x\right)/12$ the moment of inertia of the cross section about the *x*-axis. In other words, $C\left(x\right)=f_{\mathrm{res}}^{\left(\mathrm{theory}\right)}l^{2}\left(x\right)$, which is introduced for notational convenience, should be independent from *x*; that is, $C\left(x\right)=C_{0}=\left(22.37/2\pi \right){\left(Eh^{2}/12\rho \right)}^{1/2}$ m${}^{2}$· s${}^{-1}$. The experimental values of $C^{\left(i\right)}=C\left(x^{\left(i\right)}\right)$ for positions $x^{\left(i\right)}$ of the electrodes used in the present study were obtained as $C^{\left(i\right)}=\left(5.36\pm 0.16\right)\times 10^{-2}$ m${}^{2}$· s${}^{-1}$. The standard deviation was 3%, and we thus conclude that the linear theory predicts the resonant frequency of the electrodes well. Although the above discussion using beam theory is simple, the main feature of the device’s frequency selectivity is retained. Further details such as vibration mode and phase will be investigated in future work.

In Figure 3a, the amplitude was 3 nm at most, and it will be seen throughout the paper that the amplitude was on the order of 1 nm. Note that the present LDV equipment ensured a measurement of amplitude on the order of 1 nm for the frequency range from 1 to 100 kHz, which includes the frequencies treated in the present paper. According to the linear theory, we assumed that the sound-stimuli-induced vibration and the electrical-stimuli-induced vibration can be superposed when both stimuli are applied. The resonant frequencies of the device varied slightly when the device was unmounted from the experimental system and mounted again later. Therefore, the measurements of $f_{\mathrm{res}}^{\left(i\right)}$ were checked and adjusted again before each experiment. For the second, third, fourth, and fifth electrodes (which are mainly discussed in the present paper), the average resonant frequencies were, respectively, $f_{\mathrm{res}}^{\left(2\right)}=8.13\pm 0.04$ kHz, $f_{\mathrm{res}}^{\left(3\right)}=6.45\pm 0.19$ kHz, $f_{\mathrm{res}}^{\left(4\right)}=5.69\pm 0.14$ kHz, and $f_{\mathrm{res}}^{\left(5\right)}=4.55\pm 0.15$ kHz. Note that the most important frequency range in daily life is between 1 kHz and 3 kHz [36], and is lower than the resonant frequencies $f_{\mathrm{res}}^{\left(i\right)}$ obtained for the present device. Because the resonant frequency is proportional to the membrane thickness *h* as explained above, a device with a thinner membrane will be suitable for a lower hearing range, and this will be a topic of our future work. The *C* values for these four electrodes are given as $C^{\left(2\right)}=5.49\times 10^{-2}$ m${}^{2}$· s${}^{-1}$, $C^{\left(3\right)}=5.30\times 10^{-2}$ m${}^{2}$· s${}^{-1}$, $C^{\left(4\right)}=5.59\times 10^{-2}$ m${}^{2}$· s${}^{-1}$, and $C^{\left(5\right)}=5.26\times 10^{-2}$ m${}^{2}$· s${}^{-1}$. Note that these four electrodes are suitable for the validation of the proposed technique in the present paper because the first and sixth electrodes are close to the fixed ends in the *x*-direction and are affected by boundary conditions.

For vibration control using electrical stimuli, we must choose appropriate parameters ${\bar{V}}_{e}^{\left(i\right)}$, $f_{e}^{\left(i\right)}$, and $\phi^{\left(i\right)}$ contained in Equation (1) according to the sound stimuli. Here, as a test case, we determined these parameters such that the vibration of the membrane at the fifth electrode was amplified twice when sound stimuli at the resonant frequency, $f_{s}=f_{e}^{\left(5\right)}$, were applied. To be more precise, $V_{s}$ was chosen so that $a_{f}=2.5$ nm when ${\bar{V}}_{e}^{\left(5\right)}=0$ V, and ${\bar{V}}_{e}^{\left(5\right)}$ was chosen so that $a_{f}=5.0$ nm at most when both sound and electrical stimuli were applied. The frequency of electrical stimuli $f_{e}^{\left(5\right)}$ should be the same as $f_{s}$, otherwise no clear amplification or damping is expected in the present linear regime. We finally chose $\phi^{\left(5\right)}$ from the measurement. Figure 3b presents the amplitude $a_{f}$ for different phases $\phi^{\left(5\right)}$ when both sound and electrical stimuli were applied. It is clear that the amplitude was at a maximum and $a_{f}\approx 5$ nm for a certain phase $\phi^{\left(5\right)}=\phi_{\max}^{\left(5\right)}\approx 1.8\pi$, while the amplitude was at a minimum and $a_{f}\approx 0$ nm for another phase $\phi^{\left(5\right)}=\phi_{\min}^{\left(5\right)}\approx 0.8\pi$. In this manner, we can determine the phase difference of electrical stimuli for each control electrode to amplify/dampen the vibration. Note that the vibration of the membrane induced only by the sound stimuli and that only by the electrical stimuli had the same phase when there was amplification and had the opposite phase when there was damping.

Finally, we make some comments on the above experiments. It would be better if we could have predicted the values of $\phi_{\max}$ and $\phi_{\min}$ by measuring all the phase shifts in the experimental setup (e.g., by measuring the distance from the speaker and the phase shift in the electrical circuits). However, because the speaker has a typical dimension of 100 mm and it is difficult to define the distance from the speaker to the device, we measured and determined $\phi_{\max}$ and $\phi_{\min}$ experimentally. We could have attached an oscillator to the stainless-steel plate instead of the sound stimuli to induce the vibration of the membrane and to measure the piezoelectric output. However, this would have complicated the LDV analysis because we would have to distinguish the vibration of the membrane and that of the stainless-steel plate. Moreover, experiments with sound stimuli are more appropriate because our device is applied to a hearing device.

#### 3.2.2. Improvement of The Response of The Device through Vibration Control

This section presents the ability to control the vibration of the membrane by applying electrical stimuli. The amplitude was measured by the LDV for the entire range of *x* (i.e., 0 mm ≤x≤ 30 mm). In the following, ${\bar{V}}_{e}^{\left(i\right)}$ is determined such that the electrical stimuli amplify the vibration by a factor of two with $\phi^{\left(i\right)}=\phi_{\max}^{\left(i\right)}$. To be more precise, ${\bar{V}}_{e}^{\left(i\right)}$ was chosen so that $a_{f}{{|}_{x=x^{\left(i\right)}}=2a_{f}^{\prime }|}_{x=x^{\left(i\right)}}$ with $\phi^{\left(i\right)}=\phi_{\max}^{\left(i\right)}$, where $a_{f}^{\prime }{|}_{x=x^{\left(i\right)}}$ is the amplitude at $x=x^{\left(i\right)}$ with all ${\bar{V}}_{e}^{\left(i\right)}$ set to zero. The goal of this section is to amplify the vibration at the $i_{t}$-th electrode. We therefore refer to the $i_{t}$-th electrode as a target electrode. Resonant frequencies $f_{\mathrm{res}}^{\left(i\right)}$ and phases $\phi_{\min}^{\left(i\right)}$, $\phi_{\max}^{\left(i\right)}$ were measured for all electrodes, prior to the experiments described below.

(A) Only the sound stimuli with $f_{\mathrm{res}}^{\left(i_{t}\right)}$ is applied.
(B) In addition to protocol (A), electrical stimuli are applied to the $i_{t}$-th electrode with $\phi^{\left(i_{t}\right)}=\phi_{\max}^{\left(i_{t}\right)}$.
(C) In addition to protocol (B), electrical stimuli are applied to the $\left(i_{t}\pm 1\right)$-th electrode with $\phi^{\left(i_{t}\pm 1\right)}=\phi_{\min}^{\left(i_{t}\pm 1\right)}$.

Protocol (A) leads to the usual response of the device to sound stimuli. Protocol (B) tries to amplify the vibration at the resonant position. However, amplifying the vibration at the resonant position may lead to amplification of the vibration at off-resonant positions. Protocol (C) is similar to protocol (B), but the additional electrical stimuli suppress the vibration of off-resonant positions close to the resonant position, leading to better frequency selectivity. We set $i_{t}=3$, 4, and 5 and carried out each experiment five times.

**Figure 4:**
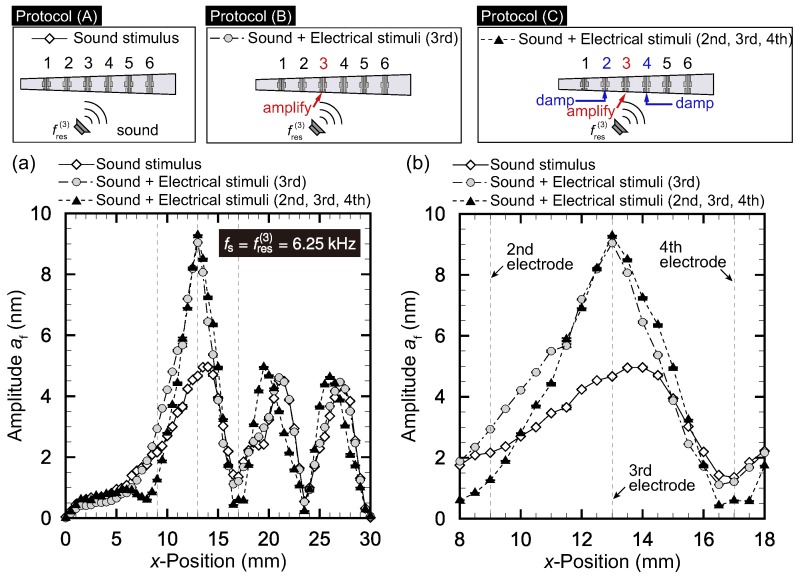
Vibration amplitude distribution for protocol (A): only sinusoidal sound stimuli with $f_{s}=6.25$ kHz; protocol (B): sound and electrical stimuli applied to the third electrode; and protocol (C): sound and electrical stimuli applied to the second to fourth electrodes. (**a**) Entire view and (**b**) magnified version near $x^{\left(3\right)}$. Protocol (B) succeeded in amplifying $a_{f}$ at resonant position $x^{\left(3\right)}=13$ mm. In addition to the amplification in Protocol (B), Protocol (C) damped $a_{f}$ of neighboring positions $x^{\left(2\right)}$ and $x^{\left(4\right)}$, where there were damping controls with opposite phases for the second and fourth electrodes.

**Figure 5:**
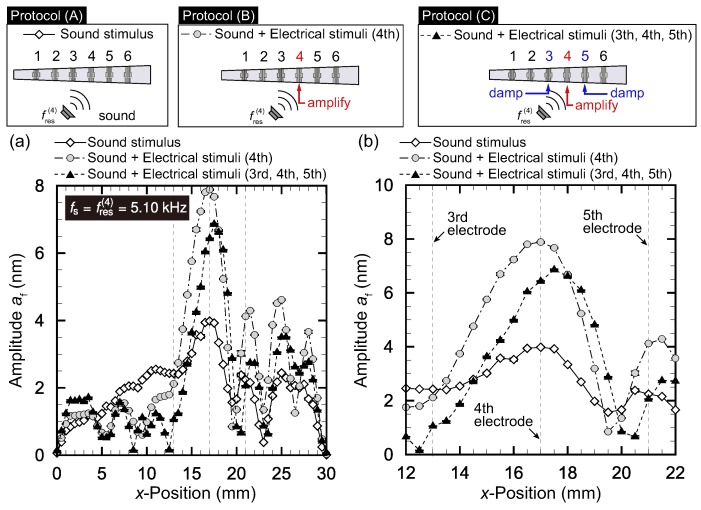
Vibration amplitude distribution for protocol (A): only sinusoidal sound stimuli with $f_{s}=5.10$ kHz; protocol (B): sound and electrical stimuli applied to the fourth electrode; and protocol (C): sound and electrical stimuli applied to the third to fifth electrodes. (**a**) Entire view and (**b**) magnified version near $x^{\left(4\right)}$. Protocol (B) succeeded in amplifying $a_{f}$ at resonant position $x^{\left(4\right)}=17$ mm. In addition to the amplification in Protocol (B), Protocol (C) suppressed $a_{f}$ of neighboring positions $x^{\left(3\right)}$ and $x^{\left(5\right)}$, where there were damping controls with opposite phases for the third and fifth electrodes.

**Figure 6:**
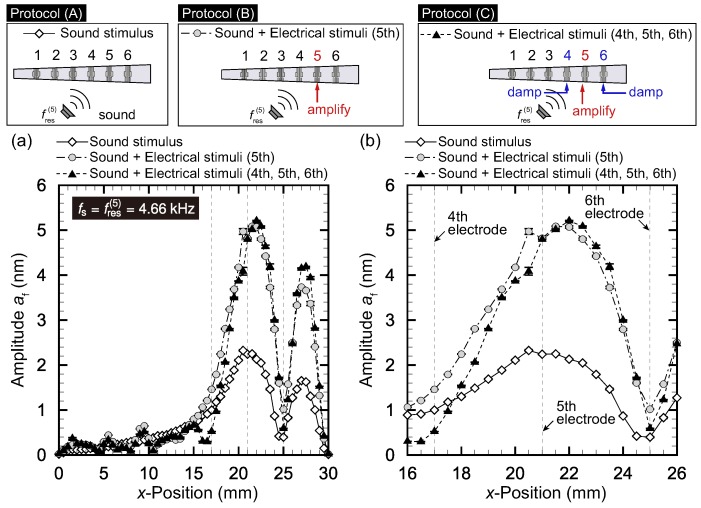
Vibration amplitude distribution for protocol (A): only sinusoidal sound stimuli with $f_{s}=4.66$ kHz; protocol (B): sound and electrical stimuli applied to the fifth electrode; and protocol (C): sound and electrical stimuli applied to the fourth to sixth electrodes. (**a**) Entire view and (**b**) magnified version near $x^{\left(5\right)}$. Protocol (B) succeeded in amplifying $a_{f}$ at resonant position $x^{\left(5\right)}=21$ mm. In addition to the amplification in Protocol (B), Protocol (C) damped $a_{f}$ of neighboring positions $x^{\left(4\right)}$ and $x^{\left(6\right)}$, where there were damping controls with opposite phases for the fourth and sixth electrodes.

The results for $i_{t}=3$, 4, and 5 are respectively presented in Figure 4, Figure 5 and Figure 6. In each figure, panel (a) shows the amplitude distribution for the entire range of *x* while panel (b) is the magnified view around $x^{\left(i_{t}\right)}$. The plots show averages for five trials with the error bars representing standard deviations.

We first focus on Figure 4, where the target electrode is the third electrode. The frequency $f_{s}$ of sound stimuli was set to the resonant frequency at the position of the third electrode, $x^{\left(3\right)}$ = 13 mm (i.e., $f_{s}=f_{\mathrm{res}}^{\left(3\right)}=6.25$ kHz). The result of protocol (A) shows that the amplitude was 4.6 nm at the electrode position of $x^{\left(3\right)}=13$ mm, which was slightly smaller than the amplitude $a_{f}=5.0$ nm at x=14 mm, even though the sound stimuli were set to the resonant frequency at x=13 mm. This is attributed to the fact that the width in the *y*-direction, l(x), was larger for x=14 mm. Note that the amplitude tended to be larger if the width l(x) was wider. Moreover, we observed local maxima at x=21 mm and x=27 mm. These local maxima were related to the oscillation mode in the *x* direction, which can also be observed in Figure 2 with $f_{s}=6.6$ kHz and in Reference [2]. At this stage, the amplitude at the resonant position was not prominent. We then tried protocol (B), where the electrical stimuli of $f_{e}^{\left(3\right)}=f_{\mathrm{res}}^{\left(3\right)}=6.25$ kHz were applied in addition to the sound stimuli. We observed an obvious amplification as a result of the electrical stimuli, and the amplitude at $x^{\left(3\right)}$ was 8.9 nm. We finally tried protocol (C), applying the electrical stimuli of $f_{e}^{\left(2\right)}=f_{e}^{\left(4\right)}=6.25$ kHz with $\phi^{\left(2\right)}=\phi_{\min}^{\left(2\right)}$ and $\phi^{\left(4\right)}=\phi_{\min}^{\left(4\right)}$. Figure 4b shows that the amplitudes at $x^{\left(2\right)}$ and $x^{\left(4\right)}$ were successfully suppressed.

Figure 5 with the target electrode being the fourth electrode shows a situation similar to that of Figure 4 with the target electrode being the third electrode. The resonant frequency was $f_{\mathrm{res}}^{\left(4\right)}=5.10$ kHz. For protocol (A), the amplitude at $x^{\left(4\right)}=17$ mm was at a maximum (4.0 nm), although we saw smaller peaks at x=20.5 mm and x=25 mm. As in the case of Figure 4, these smaller peaks must be due to the higher vibration mode at larger *x*, as also seen in Figure 2 with $f_{s}=5.6$ kHz. With the electrical stimuli at the fourth electrode in protocol (B), the amplitude at $x^{\left(4\right)}$ was amplified to 7.9 nm. However, the small peaks at x=20.5 mm and x=25 mm were also amplified. For protocol (C), where the sinusoidal electrical stimuli with opposite phase $\phi^{\left(i_{t}\pm 1\right)}=\phi_{\min}^{\left(i_{t}\pm 1\right)}$ were applied to the third and fifth electrodes, the amplitudes at $x^{\left(3\right)}$ and $x^{\left(5\right)}$ were lower, but the amplitude at $x^{\left(4\right)}$ also decreased from 7.9 to 6.7 nm.

The case where the target electrode was the fifth electrode is described in Figure 6, which should be compared with Figure 4 and Figure 5 for the target electrode being the third and fourth electrodes, respectively. The resonant frequency was $f_{\mathrm{res}}^{\left(5\right)}=4.66$ kHz. The amplitude at $x^{\left(5\right)}$ was 2.3 nm in protocol (A). As in the cases of $i_{t}=3$ and $i_{t}=4$, we observed a smaller peak at x=27 mm. The electrical stimuli of the fifth electrode in protocol (B) led to the amplification of amplitude at $x^{\left(5\right)}$, and $a_{f}=4.8$ nm was obtained. However, the smaller peak at x=27 mm was also amplified. In protocol (C), where damping control was carried out for the fourth and sixth electrodes, the amplitudes at $x^{\left(4\right)}$ and $x^{\left(6\right)}$ were lower as shown in Figure 6b.

To quantify the effect of vibration control using electrical stimuli, we define a parameter $S^{\left(i\right)}$ as

(2) $$S^{\left(i\right)}=\frac{a_{\max}^{\left(i\right)}}{x_{L}^{\left(i\right)}-x_{R}^{\left(i\right)}},$$

where $a_{\max}^{\left(i\right)}$ is the maximum amplitude for all *x*, and $x_{L}^{\left(i\right)}$ and $x_{R}^{\left(i\right)}$ are positions such that the amplitude $a_{f}$ becomes half of $a_{\max}^{\left(i\right)}$. If $S^{\left(i\right)}$ is large, the amplitude at the resonant electrode is high and localized; that is, the response of the device is improved and $S^{\left(i\right)}$ can be considered as a variant of Q factors. The response factors $S^{\left(i_{t}\right)}$ ($i_{t}=3$, 4, and 5) for the above protocols are summarized in Table 1. For $i_{t}=3$ and 5, a comparison of protocols (A) and (B) shows that $S^{\left(i_{t}\right)}$ in protocol (B) became 2.2 times that in protocol (A). This is because we set $V_{e}^{\left(i_{t}\right)}$ such that the amplitude doubled in amplifying control. For $i_{t}=4$, the increase in $S^{\left(4\right)}$ in protocol (B) was prominent, and the ratio between $S^{\left(4\right)}$ in protocols (B) and (A) took the value 1.67/0.35 = 4.77. This is because the peak near the maximum amplitude was not sharp when only sound was applied (protocol (A)), as shown in Figure 5 . We therefore conclude that vibration control worked well for better frequency selectivity, especially when the spatial response of the artificial cochlear epithelium was not sharp. The comparison between the response factor $S^{\left(i_{t}\right)}$ for protocols (B) and (C) showed that additional damping control increased $S^{\left(i_{t}\right)}$ for $i_{t}=3$ and 5, but slightly reduced for $i_{t}=4$. This decrease was due to the suppressed maximum amplitude in protocol (C) for $i_{t}=4$. To increase the $S^{\left(i_{t}\right)}$ value, it is necessary to predict the motion of the trapezoidal membrane induced by localized electrical stimuli.

The sound pressure level $P_{s}$ in the above experiments is now described. We chose the magnitude of sound stimuli ${\bar{V}}_{s}$ such that the amplitude $a_{f}$ was on the order of 1 nm, because the amplitude of the vibration of the basilar membrane typically falls in this range. For $i_{t}=3$, 4, and 5, $P_{s}$ were obtained as 92, 69, and 93 dB SPL. These $P_{s}$ are within the the wide dynamic range of human hearing from 0 to 120 dB SPL.

#### 3.2.3. Nonlinear Response of the Device to Sound

Human hearing has a dynamic range from 0 to 120 dB in amplitude, although the range of displacement of the cochlear epithelium is from 0.1 to 10 nm. Therefore, the magnitude of the pressure disturbance over $10^{6}$ times (from 20 μPa to 20 Pa) is compressed to the amplitude range over 100 times (0.1 nm to 10 nm). Such a compression is caused by the outer hair cells in the organ of Corti, which elongate and shorten according to sound stimuli to control the movement of basilar membrane [33]. In Reference [34], using a cantilever device, a nonlinear feedback control which realizes the same compression rate as the human hearing is achieved by introducing a cubic damping term with respect to the cantilever velocity in the control signal. Here, we demonstrate that the same compression rate can be achieved using our MEMS-fabricated artificial cochlea.

**Figure 7:**
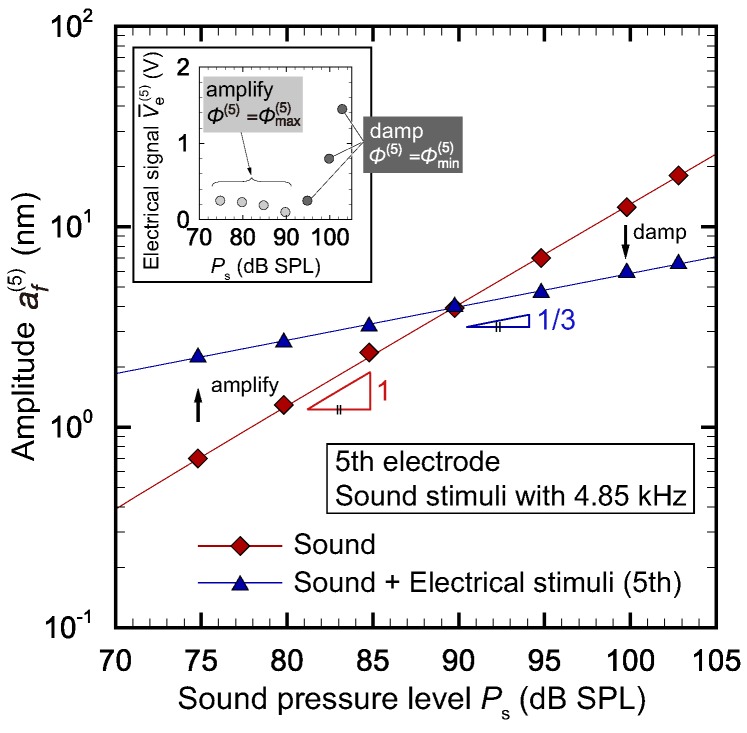
Relationship between amplitude $a_{f}$ at $x^{\left(5\right)}$ and sound pressure level $P_{s}$ for $f_{s}=4.85$ kHz. The case with sound stimuli showed a linear response, while the case with both sound and electrical stimuli showed a nonlinear response with a power 1/3. The electrical signal ${\bar{V}}_{e}^{\left(5\right)}$ used in the experiment is plotted in the inset.

Without any electrical stimuli, our device showed a linear response to the sound; that is, $a_{f}∼p$, where *p* is the magnitude of the pressure disturbance induced by sound. Figure 7 shows the amplitude at the position of the fifth electrode when a sound stimuli with resonant frequency at this position, $f_{s}=f_{\mathrm{res}}^{\left(5\right)}=4.85$ kHz, was applied. It is seen that, without electrical stimuli, the amplitude showed a linear response to the sound pressure level. Note that the sound pressure level is related to *p* as $P_{s}=20\log_{10}\left(p/p_{0}\right)$, where $p_{0}=20$ μPa is the lowest-level of human hearing. To realize the nonlinear response, we applied an electrical stimuli to the fifth electrode, where the magnitude of the electrical signal ${\bar{V}}_{e}^{\left(5\right)}$ and the phase $\phi^{\left(5\right)}$ are shown in the inset. As shown in Figure 7, the amplitude with electrical stimuli was amplified for $P_{s}\leq 90$ dB SPL and was damped for $P_{s}\geq 90$ dB SPL, realizing the nonlinear response. The lines are the fitting curves to the experimental results shown by symbols. We used $a_{f}∼p$ and $a_{f}∼p^{1/3}$ as fitting curves for the results without electrical stimuli and those with electrical stimuli, respectively. This simple demonstration shows that the present concept of mimicking outer hair cells is feasible within the range of sound pressure level investigated. However, as shown in the inset of Figure 7, electrical signal with a couple of volts are necessary to dampen large vibration. Therefore, the device performance is limited by the electrical power supply.

### 3.3. Recognition of Vibration Using Electrical Outputs

Owing to the vibration of the membrane, the *i*-th recognition electrodes generate an electrical output with amplitude ${\bar{V}}_{\mathrm{rec}}^{\left(i\right)}$. We measured ${\bar{V}}_{\mathrm{rec}}^{\left(i\right)}$ and investigated their relation to the vibration amplitude $a_{f}^{\left(i\right)}$ of the membrane and the magnitude of the sound stimuli ${\bar{V}}_{s}$ shown in Figure 1c. Note that ${\bar{V}}_{\mathrm{rec}}^{\left(i\right)}$ is proportional to the magnitude of the strain of the piezoelectric membrane, which is closely related to the vibration amplitude $a_{f}^{\left(i\right)}$.

**Figure 8:**
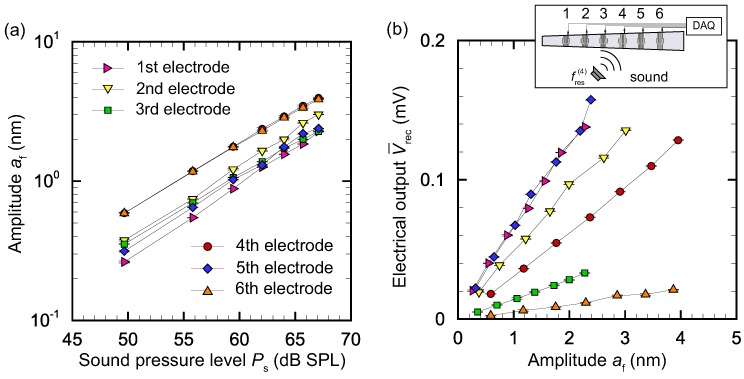
(**a**) Relationship between amplitude $a_{f}$ at $x^{\left(i\right)}$ (*i* = 1, 2, ⋯, 6) and sound pressure level $P_{s}$ for $f_{s}=f_{\mathrm{res}}^{\left(4\right)}=5.84$ kHz. The membrane vibration increased with the sound pressure level applied to the artificial cochlear epithelium. (**b**) Relationship between electrical output ${\bar{V}}_{\mathrm{rec}}^{\left(i\right)}$ (*i* = 1, 2, ⋯, 6) and amplitude $a_{f}$ at $x^{\left(i\right)}$ for $f_{s}=f_{\mathrm{res}}^{\left(4\right)}=5.84$ kHz. Amplitude $a_{f}$ and electrical output ${\bar{V}}_{\mathrm{rec}}$ from the recognition electrodes had a linear relationship.

**Figure 9:**
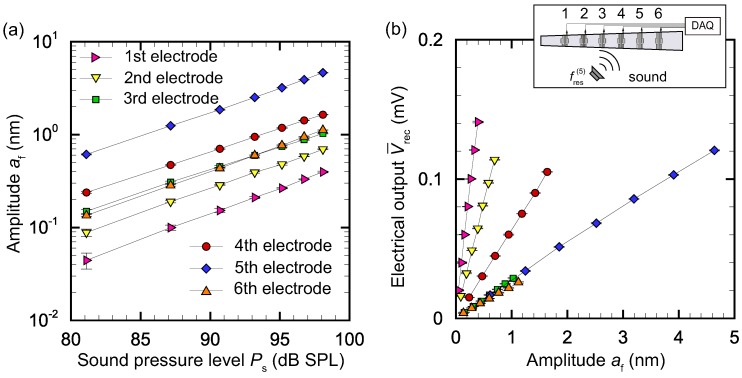
(**a**) Relationship between amplitude $a_{f}$ at $x^{\left(i\right)}$ (*i* = 1, 2, ⋯, 6) and sound pressure level $P_{s}$ for $f_{s}=f_{\mathrm{res}}^{\left(5\right)}=4.56$ kHz. The membrane vibration increased with the sound pressure level applied to the artificial cochlear epithelium; (**b**) Relationship between electrical output ${\bar{V}}_{\mathrm{rec}}^{\left(i\right)}$ (*i* = 1, 2, ⋯, 6) and amplitude $a_{f}$ at $x^{\left(i\right)}$ for $f_{s}=f_{\mathrm{res}}^{\left(5\right)}=4.56$ kHz. Amplitude $a_{f}$ and electrical output ${\bar{V}}_{\mathrm{rec}}$ from the recognition electrodes had a linear relationship.

We present results for two cases, $f_{s}=f_{\mathrm{res}}^{\left(4\right)}=5.84$ kHz and $f_{s}=f_{\mathrm{res}}^{\left(5\right)}=4.56$ kHz, and analyze ${\bar{V}}_{\mathrm{rec}}^{\left(i\right)}$ for i=1, 2, ⋯, 6. The results for these two frequencies are respectively shown in Figure 8 and Figure 9. Figure 8a shows the relationship between amplitude $a_{f}$ at $x^{\left(i\right)}$ (*i* = 1, 2, ⋯, 6) measured by the LDV and sound pressure level $P_{s}$ for $f_{s}=f_{\mathrm{res}}^{\left(4\right)}=5.84$ kHz. It is seen that the amplitude had a linear relationship with $P_{s}$, as expected. Figure 8b presents the relationship between electrical output ${\bar{V}}_{\mathrm{rec}}^{\left(i\right)}$ (*i* = 1, 2, ⋯, 6) and amplitude $a_{f}$ at $x^{\left(i\right)}$ for $f_{s}=f_{\mathrm{res}}^{\left(4\right)}=5.84$ kHz. ${\bar{V}}_{\mathrm{rec}}^{\left(i\right)}$ is linearly correlated with $a_{f}^{\left(i\right)}$. However, the coefficients of proportionality depend on the electrode; that is, when we define a displacement–output conversion factor $\gamma^{\left(i\right)}={\bar{V}}_{\mathrm{rec}}^{\left(i\right)}/a_{f}^{\left(i\right)}$ (V/m), $\gamma^{\left(i\right)}$ changes according to *i*. The conversion factor $\gamma^{\left(i\right)}$ tends to be smaller for larger $x^{\left(i\right)}$. This is because the same amplitude results in larger deformation of the membrane when l(x) is smaller. In Section 3.4, we use $V_{\mathrm{rec}}^{\left(i\right)}$ to recognize the electrode with maximum amplitude. The values of $\gamma^{\left(i\right)}$ are necessary for correct displacement–output conversion and feedback control.

Figure 9a confirms the linear relationship between amplitude $a_{f}^{\left(i\right)}$ and $P_{s}$ for $f_{s}=f_{\mathrm{res}}^{\left(5\right)}=4.56$ kHz; that is, the magnitude of the membrane vibration is proportional to that of sound stimuli. Figure 9b clearly shows that ${\bar{V}}_{\mathrm{rec}}^{\left(i\right)}=\gamma^{\left(i\right)}a_{f}^{\left(i\right)}$, but the values of $\gamma^{\left(i\right)}$ are different from those in Figure 8b. For $f_{s}=f_{\mathrm{res}}^{\left(5\right)}=4.56$ kHz, the conversion factor $\gamma^{\left(i\right)}$ tends to be smaller for larger $x^{\left(i\right)}$, as in the case of $f_{s}=f_{\mathrm{res}}^{\left(4\right)}=5.84$ kHz. This indicates that the amplitude $a_{f}^{\left(i\right)}$ and ${\bar{V}}_{\mathrm{rec}}^{\left(i\right)}$ have a non-trivial relationship. Further studies on the dimensions of the electrode and strain field are needed to clarify this relationship. This topic is left as future work, because the main goal of the present paper is to show the feasibility of vibration control using electrical stimuli.

### 3.4. Mimicking the Function of Outer Hair Cells by Electrical Feedback Control

**Figure 10:**
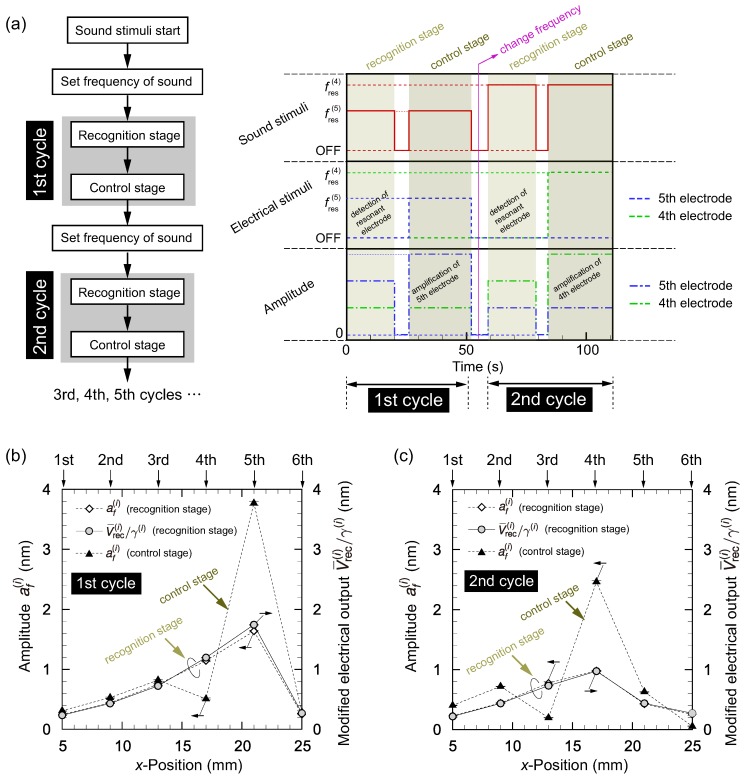
(**a**) Schematic diagram of the feedback control experiment. The experiments comprised recognition and control stages. We carried out two cycles of these stages sequentially as shown in Figure 10a. In the first and second cycles, sound stimuli with $f_{s}=f_{\mathrm{res}}^{\left(5\right)}$ and $f_{s}=f_{\mathrm{res}}^{\left(4\right)}$ were applied, respectively. The frequency of sound was changed between the first and second cycles to demonstrate that the present system responds to the frequency change. In the recognition stage, the electrical output ${\bar{V}}_{\mathrm{rec}}^{\left(i\right)}$ was automatically measured to find the resonant position. In the control stage, the electrical stimuli were applied to amplify the vibration of the resonant position found in the recognition stage. Amplitude distribution in (**b**) the first cycle and (**c**) the second cycle. In the first cycle shown in panel (b), the fifth electrode was detected as the resonant position in the recognition stage, and the amplitude of vibration was amplified only near the fifth electrode in the control stage. In the second cycle shown in panel (c), the fourth electrode was detected as the resonant position in the recognition stage, and the amplitude of vibration was amplified only near the fourth electrode in the control stage.

This section describes the results of feedback control of vibration by applying electrical stimuli to the control electrodes and using the electrical output from the recognition electrodes, which are presented in Figure 1a. A schematic diagram of the experiment is described in Figure 10a. The parameters of the electrical stimuli, such as $\phi^{\left(i\right)}$, were determined before the experiment in the same manner as in Section 3.2.2. As described in Section 3.3, it was necessary to determine $\gamma^{\left(i\right)}$ to relate the output voltage ${\bar{V}}_{\mathrm{rec}}^{\left(i\right)}$ from the recognition electrodes and the amplitude $a_{f}^{\left(i\right)}$. These values of $\gamma^{\left(i\right)}$ for each electrode and for each frequency were obtained prior to the following experiment.

The experiment comprised recognition and control stages. We carried out two cycles of these stages sequentially, as shown in Figure 10a. In the first and second cycles, sound stimuli with $f_{s}=f_{\mathrm{res}}^{\left(5\right)}$ and $f_{s}=f_{\mathrm{res}}^{\left(4\right)}$ were applied, respectively. The frequency of sound was changed between the first and second cycles to demonstrate that the present system responded to the frequency change. As a comprehensible demonstration, we chose $f_{\mathrm{res}}^{\left(5\right)}$ for the first cycle and $f_{\mathrm{res}}^{\left(4\right)}$ for the second cycle, but other frequencies can be chosen. In the recognition stage, no electrical stimuli were applied and electrical measurements of ${\bar{V}}_{\mathrm{rec}}^{\left(i\right)}$ were automatically made to find the resonant position. In the present demonstration, these resonant positions for first and second cycles were respectively $x^{\left(5\right)}$ and $x^{\left(4\right)}$. Note that the LDV measurements were also made to confirm that the amplitude was magnified/damped as expected, but were not used for feedback control. Each stage took tens of seconds because the *x*–*y* auto-stage moved at a speed of 7 mm·s${}^{-1}$ to the measurement position (i.e., $x^{\left(i\right)}$, i=1, 2, ⋯, 6) and waited five seconds before the measurement of vibration. The purpose of the present study was to develop and confirm a prototype principle of mimicking the outer hair cell as a first step. This waiting time was necessary for precise measurement because any tiny oscillation may affect the result. In the control stage, electrical stimuli were applied to amplify the vibration of the resonant position found in the recognition stage. As in protocol (C) described in Section 3.2.2, damping control was also carried out for neighboring electrodes. The results of the first and second cycles are respectively shown in Figure 10b,c. In the recognition stage of the first cycle, the position of the fifth electrode was detected as the resonant position as seen from the values of the modified electrical output ${\bar{V}}_{\mathrm{rec}}^{\left(i\right)}/\gamma^{\left(i\right)}\left(=a_{f}^{\left(i\right)}\right)$. The electrical stimuli were then applied in the control stage of the first cycle to yield a prominent peak at the fifth position. Between the first and second cycles (t≈ 50 s), the frequency of sound changed to $f_{\mathrm{res}}^{\left(4\right)}$. The measurement of electrical outputs in the recognition stage of the second cycle yielded that ${\bar{V}}_{\mathrm{rec}}^{\left(4\right)}/\gamma^{\left(4\right)}$ was the maximum and the position of the fourth electrode was thus detected as a resonant position. In the control stage of the second cycle, we successfully amplified the vibration at the fourth electrode while suppressing amplification at the neighboring electrodes (third and fifth), although the fifth electrode was slightly amplified.

We define another response factor $Q^{\left(i\right)}$ as

(3) $$\begin{array}{c} Q^{\left(i\right)}=\frac{a_{f}^{\left(i\right)}}{(a_{f}^{\left(i+1\right)}+a_{f}^{\left(i-1\right)})/2}. \end{array}$$

In the above four experiments, for the first/second cycles and recognition/control states, $Q^{\left(i\right)}$ values were obtained as given in Table 2. The table shows that the $Q^{\left(i\right)}$ value was higher in the control stage and the magnification ratios were 4.03 and 3.64 for the first and second cycles, respectively. These results indicate that the present experimental system was capable of increasing the performance of frequency selectivity of the artificial cochlear epithelium by mimicking the function of outer hair cells.

## 4. Conclusions

We propose an artificial cochlear epithelium which mimics the function of an outer hair cell using feedback electrical stimuli. The main outcomes of the present paper are summarized as follows.

1. On the basis of a previous device [2], we developed a new design of an artificial cochlear epithelium with recognition and control electrodes. These electrodes are used to mimic the functions of the basilar membrane, inner hair cells, and outer hair cells.
2. Recognition of the resonant position and control of the vibration amplitude at the resonant position are realized using the electrode pattern of the present device. The method uses the local electrical stimuli through patterned electrodes fabricated on a PVDF membrane with a trapezoidal support. Parameters of the electrical stimuli were experimentally determined for each electrode to improve the response of the artificial cochlear epithelium.
3. A demonstration of the feedback control of membrane vibration was carried out by alternating the frequency of sound stimuli during a single run of the experiment. The present device automatically responds to a change in the sound frequency and amplifies the vibration amplitude at the resonant position.

There are ways to improve the present control method. Firstly, it is important to design the recognition electrodes to evaluate the amplitude of membrane vibration quantitatively. That is to say, one needs to control values of the displacement–output conversion factor of the *i*-th electrode (i.e., $\gamma^{\left(i\right)}$ in Section 3.3) by changing the dimensions of the recognition electrodes. As an alternative solution, one may use a machine-learning technique to search for appropriate control parameters of electrical stimuli (e.g., $\phi^{\left(i\right)}$) and to construct a database of $\gamma^{\left(i\right)}$. Another important direction of improvement is minimization of the experimental setup. We constructed an automation system for the present study, but the system obviously cannot be integrated with an actual artificial cochlea. It is necessary to develop an equivalent circuit system using micro-fabrication technologies for further investigation, such as animal tests. To sustain electrical power to activate devices, one may consider using an endocochlear potential maintained in the cochlea [37,38], which has also been proposed as a biological battery [39]. The cochlear shape is important in low-frequency hearing [40,41], and thus evaluation of the device in an environment similar to that of the cochlea is also necessary for the optimal design of wide-range frequency selectivity.

## Figures and Tables

**Table 1:** Response factor $S^{\left(i_{t}\right)}$ for protocols (A), (B), and (C) with $i_{t}=3$, 4, and 5.

	$S^{\left(i_{t}\right)}$ $\times 10^{6}$		
	Protocol (A)	Protocol (B)	Protocol (C)
Sound Stimuli	On	On	On
Electrical Stimuli Applied to the $i_{t}$-th Electrode for Amplification Control	Off	On	On
Electrical Stimuli Applied to the $\left(i_{t}\pm 1\right)$-th Electrodes for Damping Control	Off	Off	On
$i_{t}=3$	0.90	2.00	2.42
$i_{t}=4$	0.35	1.67	1.53
$i_{t}=5$	0.42	0.91	0.99

**Table 2:** Response factor $Q^{\left(i\right)}$ for recognition/control stages of the first/second cycles presented in Figure 10.

	$Q^{\left(i\right)}$		$\frac{Q^{\left(i\right)}\;\mathrm{of}\;\mathrm{Recognition}\;\mathrm{Stage}}{Q^{\left(i\right)}\;\mathrm{of}\;\mathrm{Control}\;\mathrm{Stage}}$
	Recognition Stage	Control Stage	
First Cycle $\left(i_{t}=5\right)$	2.35 ± 0.06	9.48 ± 0.49	4.03
Second Cycle $\left(i_{t}=4\right)$	1.64 ± 0.03	5.96 ± 0.15	3.64

**Figure A1:**
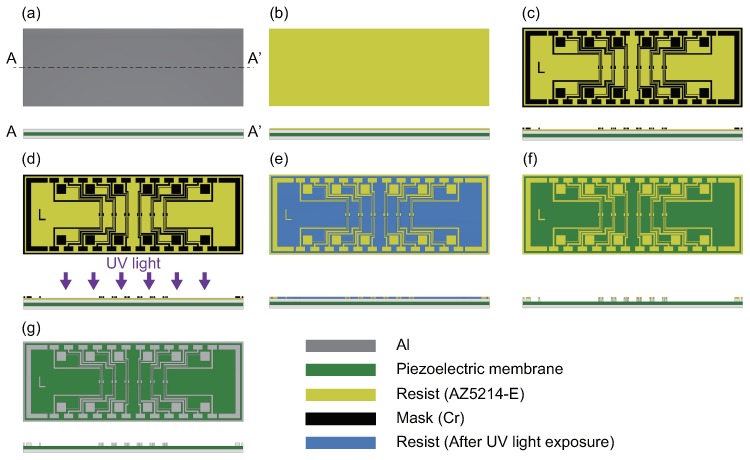
Fabrication process of the PVDF membrane with patterned electrodes. (**a**) Al-deposited piezoelectric membrane; (**b**) Spincoating of photoresist AZ5214-E; (**c**) Covering the photomask; (**d**) Exposure to ultraviolet light; (**e**) Removing the photomask; (**f**) Development and etching; (**g**) Lifting off.

## Appendix A. Fabrication of the PVDF Membrane with Patterned Electrodes

The fabrication method of the patterned electrode on the PVDF membrane is described as follows and summarized in Figure A1. An Al thin film with thickness of 50–60 nm was deposited on both sides of a PVDF membrane with thickness of 40 µm (KF piezo film, Kureha, Tokyo, Japan). The PVDF membrane was attached to a glass substrate for the following photolithography process. A positive photoresist (AZ5214E, Merck, Darmstadt, Germany) was spin-coated on the PVDF membrane at 3000 rpm. After a prebake for 10 min at 80 degrees, the membrane was exposed to ultraviolet light at 6 mJ/cm${}^{2}$ through a photomask. To expose the membrane uniformly, three glass diffuser plates were placed between the light source and membrane. The exposed photoresist was developed by immersing the membrane in developer solution (AZ300MIF, Merck, Darmstadt, Germany) for 8 min. During the development process, the Al film except for the area covered by the cured photoresist was etched, and the PVDF membrane with a patterned electrode was thus obtained as shown in Figure 1b. Finally, the membrane was immersed in ethanol for 2 min to remove the photoresist. The Al thin film on the backside of the PVDF membrane is protected and maintained by the glass substrate.
